# Survival potential of wild type cellulose deficient *Salmonella *from the feed industry

**DOI:** 10.1186/1746-6148-5-43

**Published:** 2009-11-23

**Authors:** Lene K Vestby, Trond Møretrø, Simon Ballance, Solveig Langsrud, Live L Nesse

**Affiliations:** 1 National Veterinary Institute, Section of Bacteriology, Oslo, Norway; 2 Nofima Mat, Aas, Norway

## Abstract

**Background:**

Biofilm has been shown to be one way for *Salmonella *to persist in the feed factory environment. Matrix components, such as fimbriae and cellulose, have been suggested to play an important role in the survival of *Salmonella *in the environment. Multicellular behaviour by *Salmonella *is often categorized according to colony morphology into rdar (red, dry and rough) expressing curli fimbriae and cellulose, bdar (brown, dry and rough) expressing curli fimbriae and pdar (pink, dry and rough) expressing cellulose.

The aim of the study was to look into the distribution of morphotypes among feed and fish meal factory strains of *Salmonella*, with emphasis on potential differences between morphotypes with regards to survival in the feed factory environment.

**Results:**

When screening a total of 148 *Salmonella *ser. Agona, *Salmonella *ser. Montevideo, *Salmonella *ser. Senftenberg and *Salmonella *ser. Typhimurium strains of feed factory, human clinical and reference collection origin, as many as 99% were able to express rough morphology (rdar or bdar). The dominant morphotype was rdar (74%), however as many as 55% of *Salmonella *ser. Agona and 19% of *Salmonella *ser. Senftenberg displayed the bdar morphology.

Inconsistency in Calcofluor binding, indicating expression of cellulose, was found among 25% of all the strains tested, however *Salmonella *ser. Agona showed to be highly consistent in Calcofluor binding (98%).

In biofilm, *Salmonella *ser. Agona strains with bdar mophology was found to be equally tolerant to disinfection treatment as strains with rdar morphotype. However, rdar morphology appeared to be favourable in long term survival in biofilm in a very dry environment.

Chemical analysis showed no major differences in polysaccharide content between bdar and rdar strains. Our results indicate that cellulose is not a major component of the *Salmonella *biofilm matrix.

**Conclusion:**

The bdar morphotype is common among *Salmonella *ser. Agona strains isolated from the factory environment. The rdar and the bdar strains were found to be equally tolerant to disinfectants, while the rdar strain was found to be more tolerant to long-term desiccation and nutrient depletion in biofilm than the bdar strain. Cellulose does not appear to be a major component of the *Salmonella *biofilm matrix.

## Background

*Salmonella *is a well known contaminant of feed and the feed industry, and the authorities and the industry are using large resources in the fight against *Salmonella *[1]. Despite this, *Salmonella *is still isolated from several different control points in the Norwegian feed and fish meal factory environments and some clones have even been shown to persist in these environments for several years [2-4]. Biofilm has been shown to be a way for *Salmonella *to persist in the feed and fish meal factory environment [5].

Biofilms are defined as matrix-enclosed bacterial populations adherent to each other and/or surfaces or interfaces [6,7]. Several physiological changes like altered growth rate and transcription of different genes have been observed among bacteria in biofilms [8]. Biofilms are both medically and industrially important because they can accumulate on a wide variety of substrates, and bacteria in biofilms are more resistant to antimicrobial agents than their planktonic counterparts [7,9,10].

Previous studies have indicated that *Salmonella *matrix components, such as fimbriae and cellulose, play an important role in the survival of *Salmonella *in the environment [11-13]. Cellulose is the most abundant biopolymer in nature. It is produced by various species including plants, animals, algae and bacteria [14]. While plants produce cellulose as a structural component of the cell wall, bacteria generally produce cellulose as an extracellular component for mechanical and chemical protection [15]. Multicellular behaviour based on matrix components by *Salmonella *is often categorised into three groups according to colony morphology when grown on agar plates containing Congo Red and Coomassie brilliant blue dye; rdar (red, dry and rough), bdar (brown, dry and rough) and pdar (pink dry and rough). Colonies without multicellular morphology are called saw (smooth and white). Strains with rdar morphology express both thin aggregative fimbriae (curli) and cellulose, those with bdar morphology express only curli fimbriae and not cellulose, those with pdar express only cellulose and not curli fimbriae whilst those with saw morphology expresses neither [16-18].

Although a few screening studies on different serovars have been reported, most studies on multicellular behaviour by *Salmonella *have been performed on *Salmonella enterica *subspecies *enterica *serovar Typhimurium using reference collection strains and constructed knockout mutants. Several studies have been performed on the composition of the matrix on rdar morphotypes [13,16,17,19-21], but not much focus has been given the bdar morphotype in wild type strains.

The aim of this study was to look into the distribution and matrix composition of the bdar morphotype among feed and fish meal factory *Salmonella *strains with emphasis on potential differences between the rdar and the bdar strains' ability to survive in this environment. All investigations were conducted on strains from Norwegian feed and fish meal factories of *Salmonella *ser. Agona, *Salmonella *ser. Montevideo, *Salmonella *ser. Senftenberg and *Salmonella *ser. Typhimurium along with strains from human clinical cases and reference collection strains. *Salmonella *ser. Agona, *Salmonella *ser. Montevideo and *Salmonella *ser. Senftenberg are frequently isolated from the feed industry, while *Salmonella *ser. Typhimurium was included due to this serovar being endemic in Norwegian wild life and is one of the most thoroughly studied *Salmonella *serovars in biofilm experiments.

## Methods

### Bacterial strains and culture conditions

A total of 148 *Salmonella *strains of serovar Agona, serovar Montevideo, serovar Senftenberg and serovar Typhimurium were used in this study (Table 1). These included strains of feed factory, clinical and reference collection origin. All isolations were carried out at different private or official laboratories and verified at the National Reference Laboratory for *Salmonella *in Feed and Food at the National Veterinary Institute.

**Table 1:** Number of strains of different serovars and sources

Serovar	Factory strains	Human strains	Reference strains^§^
Agona	38	8	1
Montevideo	29	8	1
Senftenberg	33	8	1
Typhimurium	11	8	2

^§ ^*Salmonella *ser. Agona FHBA266, *Salmonella *ser. Montevideo FHBA46, *Salmonella *ser. Senftenberg FHBA87, *Salmonella *ser. Typhimurium ATCC 14028, *Salmonella *ser. Typhimurium ATCC 2700720D (LT2)

All strains were stored at -80°C in Brain Heart Infusion broth (BHI; Difco, BD, Franklin Lakes, NJ, USA) supplemented with 15% glycerol (Merck KGaA, Darmstadt, Germany) and recovered on blood agar (sheep blood) at 37.0 ± 1.0°C overnight. The bacterial cultures were then transferred into Luria Bertani broth (LB; Merck) and incubated statically overnight at 37.0 ± 1.0°C to obtain an overnight working culture.

### Screening of morphotype

All 148 strains were screened at least twice for colony morphology using previously described methods with slight modifications [22]. In brief; colonies were inoculated onto LB agar without NaCl (bacto-tryptone 10 g/L, yeast extract 5 g/L, agar 15 g/L) containing 40 μg/mL Congo Red (Merck) and 20 μg/mL Coomassie brilliant blue (Sigma-Aldrich, St. Louis, MO) (CR agar). After inoculation, the CR agar plates were incubated at 20.0 ± 1.0°C for up to eight days. All plates were visually examined and the morphotypes were categorised as: rdar - indicating expression of curli fimbriae and cellulose, bdar - indicating expression of fimbriae but not cellulose, pdar - indicating expression of cellulose but not fimbriae and saw - indicating expression of neither cellulose nor fimbriae.

### Screening of Calcofluor binding

All 148 strains were screened at least twice on agar and liquid-air interface to investigate cellulose production as indicated by Calcofluor (fluorescent brightener 28, Sigma-Aldrich) binding. On agar, a previously described method with slight modifications was used [22]. In brief; colonies were inoculated onto LB agar without NaCl containing 200 μg/mL Calcofluor (CF agar), incubated at 20.0 ± 1.0°C for eight days, and visually examined after two, four, six and eight days. Fluorescing colonies when exposed to UV light indicated the presence of cellulose. A modified pellicle assay was developed in order to study indicated presence of cellulose in the pellicle in the liquid-air interface [11]. Pellicle formation was investigated using 4.5 mL LB ^wo^/NaCl containing 200 μg/mL Calcofluor (Sigma-Aldrich) inoculated with 0.5 mL of an overnight culture, and incubated statically at 20.0 ± 1.0°C for up to eight days. The strains were visually examined and categorized according to pellicle formation or not. Fluorescing of the pellicle when exposed to UV light indicated the presence of cellulose. Selected strains were also tested for calcofluor binding on glass, stainless steel and polystyrene surfaces (Nunc, Nuncleon, Roskilde, Denmark), either by biofilm formation on coupons (Stainless steel, AISI 304,2B, 7 by 2 by 0.1 cm pieces) partially submerged in LB ^wo^/NaCl containing 200 μg/mL Calcofluor (Sigma-Aldrich) or biofilm formation on the walls of reagents tubes (glass or polystyrene surfaces). Samples were incubated statically at 20.0 ± 1.0°C for eight days before visual examination under UV light. Consistency in Calcofluor binding was defined as strains that always were able to bind Calcofluor when tested repeatedly on different or same type of surface.

### Crystal violet binding assay

The crystal violet binding assay was performed in microtiter plates as earlier described [5]. In brief; overnight cultures were diluted in LB broth ^wo^/NaCl to OD595 = 0.2, and 30 μL of this suspension were transferred to each well in 96 wells polystyrene microtiter plates (Nunc, Nuncleon) containing 100 μL LB broth ^wo^/NaCl (three parallels of each strain), and the microtiter plates were incubated statically for two days at 20.0 ± 1.0°C. When testing the effect of prolonged incubation, the plates were incubated statically at 20.0 ± 1.0°C for one, two, three and four days. After incubation, OD_595 _were measured before the plates were gently washed once with sterile distilled water (SDW). The plates were dried in room temperature before addition of 130 μL 1% crystal violet (Sigma). After 30 minutes incubation in room temperature, the plates were washed three times with SDW before addition of 130 μL ethanol:acetone (70:30 v:v) and incubation for 10 minutes in room temperature. OD_595 _were measured after the bound dye was dissolved using ethanol and acetone. For each strain, the result was calculated by subtracting the median OD_595 _of the three parallels of the control (test broth only) from the median OD_595 _of the three parallels of sample.

### Polysaccharide analysis

#### Rdar and bdar matrix from pellicles

For obtaining pellicles for analysis of polysaccharide, LB ^wo^/NaCl was inoculated with wild type *Salmonella *ser. Agona strains 71-3 (rdar) and 1454-1 (bdar) along with *Salmonella *ser. Typhimurium reference collection strains ATCC 14028 (rdar) and ATCC 2700720D (LT-2) (rdar) cultivated on agar plates. *Acetobacter xylinum *30.3 (kind gift from Svein Valla, Norwegian University of Science and Technology) was used a positive control of cellulose production. *A. xylinum *was cultivated in Hestrin and Schramm medium (glucose 20 g/L, yeast extract 5 g/L, peptone 5 g/L, Na_2_HPO_4 _2.7 g/L, citric acid 1.15 g/L, pH adjusted to 6.0) [23] at 30.0 ± 1.0°C. To obtain large pellicles the strains were cultivated in beakers with 100-200 mL medium at 25.0 ± 1.0°C and 30.0 ± 1.0°C for the *Salmonella *strains and *A. xylinum*, respectively. After 7-10 days the pellicles were harvested with a bacterial loop and transferred to beakers with 300 mL distilled water. Within 10 min the pellicles were transferred to test tubes and frozen at - 20.0 ± 1.0°C, followed by freeze drying within two days. Samples were stored under vacuum over P_2_O_5 _prior to further analysis.

Isolation of cellulose from pellicles was obtained by incubation for 30 min at 100.0 ± 1.0°C of 100-200 mg pellicle in 8:2:1 acetic acid: nitric acid: distilled water at a ratio of 3 mL per 200 mg dry weight [24,25]. A sample of 85% pure cellulose was used as a positive control. Following hydrolysis, the mixture was centrifuged for 10 min at 4000 *g *to pellet any cellulose. The supernatant was discarded. The pellet was then re-suspended in water, thoroughly washed via vortexing, and re-pelleted via centrifugation. This process was repeated five times where water was replaced with EtOH and acetone in the fourth and fifth round respectively. The pellet was then dried in an oven overnight at 50.0 ± 1.0°C prior to analysis of its monosaccharide composition described below.

#### Rdar and bdar matrix from biofilms on agar

For wild type *Salmonella *ser. Agona strains rdar 71-3 and bdar 1454-1 production of extracellular biofilm polysaccharides was also examined for colonies grown on agar. Extracellular polysaccharides were isolated according to methods described by Azeredo *et al*. (1999) [26]. Cells grown for 7-10 days on LB agar ^wo^/NaCl at 25 ± 1°C, were harvested by scraping the cells with a microscope slide. Aliquots of 1.5 mL of 0.2 M phosphate buffer (pH 7) was added to the cell mass and the sample sonicated (2 min on ice, Microson ultrasonic cell disruptor), vortexed and centrifuged. All samples where placed on ice between each step. The supernatant was pooled into a new tube, added 2 volumes of cold 96% w/v EtOH and stored overnight at 4.0 ± 1.0°C. The precipitated polysaccharide was collected by centrifugation (15,000 *g*, 20 min, 4.0 ± 1.0°C). The pellet was then washed with EtOH twice and re-pelleted each time before freeze-drying.

#### Analysis of monosaccharide composition

Freeze-dried samples were stored over P_2_O_5 _for a few days. Equivalents of 5-15 mg were weighed out into a hydrolysis tube. The sample was then incubated in 0.5 mL 12 M H_2_SO_4 _for 30 min at 35.0 ± 1.0°C followed by addition of 2.5 mL water to make the solution 2 M H_2_SO_4 _and incubated in a boiling water bath for one hour. The liberated sugars were then converted into their corresponding alditol acetates and quantified via monosaccharide standards as described by Englyst *et al*. (1994) [27]. In addition the cellulose content of extracellular polysaccharides isolated from 71-3 and 1454-1 was estimated calculating the difference in glucose content between samples treated with or without 12 M H_2_SO_4 _[27]. Alditol acetates were analysed on a DB-23 silica capillary column using flame ionization detection on an Agilent GC. Data was collected and processed using Chemstation software. All values are expressed as grams polysaccharide per 100 g sample (oven-dried) weight. A universal multiplication factor of 0.89 was used to convert from monosaccharide to polysaccharide.

### Survival of rdar and bdar strains in biofilm

#### Effect of disinfectants on rdar and bdar strains in biofilm

Three *Salmonella *ser. Agona strains displaying rdar morphology (71-3, 2168-10, 1651-7) and three *Salmonella *ser. Agona strains displaying bdar morphology (1454-1, 71-4, 1825-11) were used to evaluate the effect of 0.05% hypochlorite (Klorin; Lilleborg AS, Oslo, Norway) and 0.02% benzalkonium chloride (Norwegian Pharmaceutical Depot, Oslo, Norway) on *Salmonella *in biofilm. Overnight cultures were inoculated (1 μL) in sterile centrifuge tubes (Greiner bio-one GmbH) containing 10 mL LB ^wo^/NaCl. An autoclaved microscope slide (Menzel GmbH + CoKG) was placed in each tube. The tubes were incubated at 20.0 ± 1.0°C for two days. The slides were washed in sterile saline before drying in room temperature in a safety hood for approximately one hour. The bactericidal effect of disinfectants was tested by submerging the slides in 15 mL disinfectant solution for five minutes at 20.0 ± 1.0°C followed by submersion in 20 mL Dey/Engley neutralising broth (Difco). Controls were treated in the same way, but with sterile saline. The biofilm was thoroughly scraped using a sterile cell scraper (BD Falcon) and transferred to reagent tubes containing 4 mL sterile saline and about 30 (3 mm) sterile glass beads. The biofilm was disrupted by vortexing at maximum speed for 40 s before parallel serial dilution and plating onto blood agar (sheep blood) before incubating overnight at 37.0 ± 1.0°C to enumerate the total number of CFU in the biofilm [10,28]. Duplicate samples were used, and two to six independent experiments were performed.

#### Long-term survival in biofilm

Overnight cultures of *Salmonella *ser. Agona 71-3 (rdar) and *Salmonella *ser. Agona 1454-1 (bdar), both isolated from feed factories, were inoculated (1 μL) in sterile centrifuge tubes (Greiner bio-one GmbH, Frickenhausen, Germany) containing 10 mL LB ^wo^/NaCl. An autoclaved microscope slide (76 × 26 mm, Menzel GmbH + CoKG, Braunschweig, Germany) was placed in each tube. The tubes were incubated at 20.0 ± 1.0°C for two days. During incubation, biofilm was formed on both sides of the microscope slide at the liquid-air interface. The slides were washed in sterile saline before drying for approximately one hour in room temperature. The slides for long-term survival were placed in an empty centrifuge tube for approximately four months followed by submersion in 15 mL sterile saline. The biofilm was thoroughly scraped using a sterile cell scraper (BD Falcon, Bedford, MA, USA) and transferred to reagent tubes containing 4 mL sterile saline and about 30 (3 mm) sterile glass beads. The biofilm was disrupted by vortexing at maximum speed for 40 s before parallel serial dilution and plating onto blood agar (sheep blood) before incubation overnight at 37.0 ± 1.0°C to enumerate the total number of CFU in the biofilm [10,28]. Triplicate samples were used and the results were log_10_- transformed. The experiment was performed twice with three parallels.

#### Statistics

Statistical analyses were performed using the software JMP (SAS institute Inc. version 5.01a, Cary, NC, USA). For statistical analysis of survival of *Salmonella *in biofilm and effect of disinfectants on rdar and bdar strains, log_10 _transformed values were used. Statistical methods are given under results.

## Results

### The prevalence of bdar morphology

**Figure 1:**
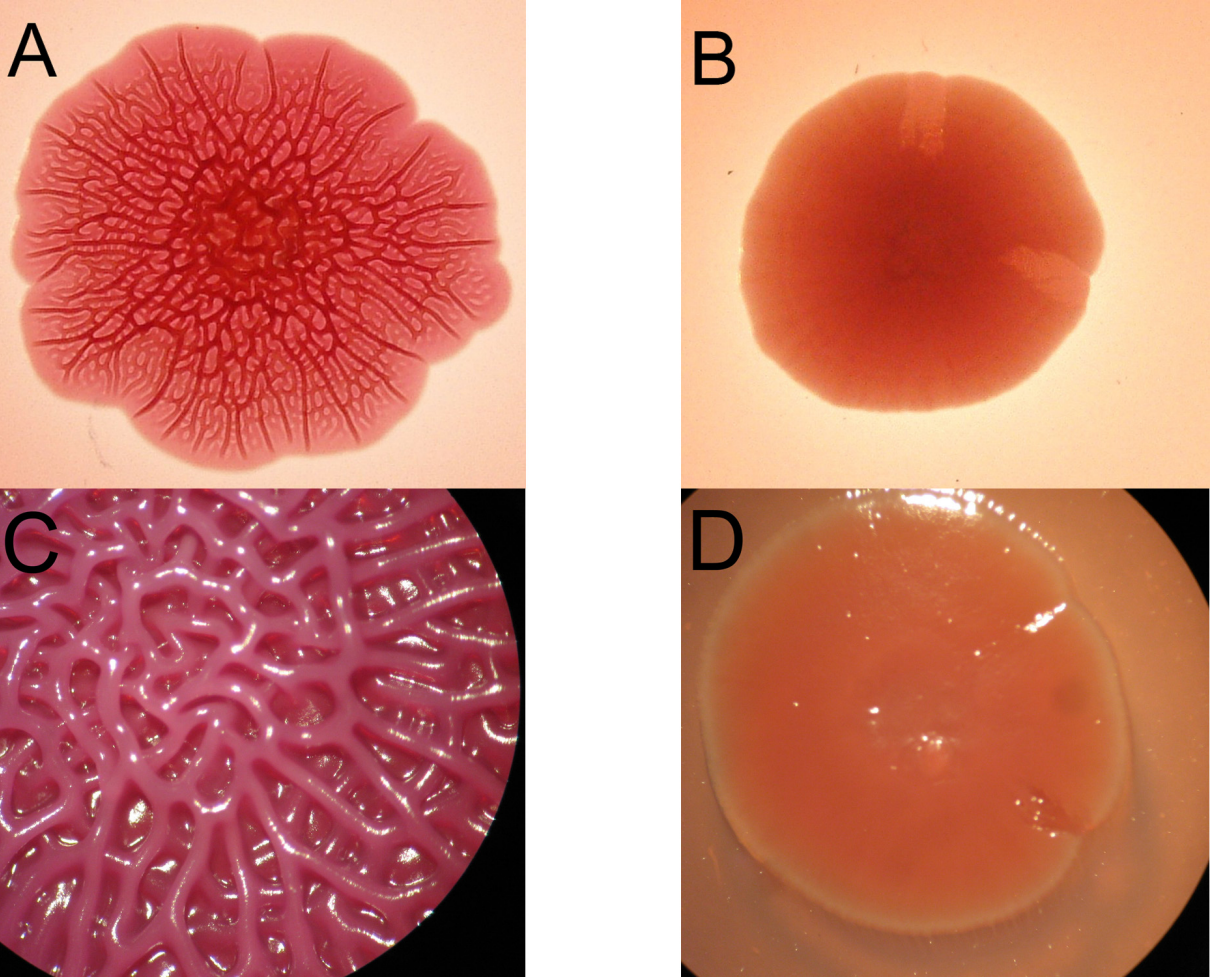
***Salmonella *rdar and bdar morphotypes**. Rdar (red, dry and rough) morphology by *Salmonella *ser. Agona strain 71-3 close up (A) and through a magnifying glass (C), and bdar (brown, dry and rough) morphology by *Salmonella *ser. Agona strain 1454-1 close up (B) nd through a magnifying glass (D), as seen on CR agar after incubation at 20°C.

When screening all strains at 20°C, a total of 99% displayed rough morphology after eight days. The dominant biofilm morphotype was rdar (red, dry and rough) (74%) (Figure 1). However, as many as 61% of *Salmonella *ser. Agona factory strains and 21% of *Salmonella *ser. Senftenberg factory strains expressed the bdar (brown, dry and rough) morphotype (Table 2, Figure 1). Among the human strains, this morphotype was expressed by three out of eight *Salmonella *ser. Agona strains (38%) and one out of eight *Salmonella *ser. Senftenberg strains (13%).

**Table 2:** Number of different morphotypes within each serovar

Serovar	Rdar			Bdar			Saw		
	
	Feed factory	Human	Reference collection	Feed factory	Human	Reference collection	Feed factory	Human	Reference collection
Agona	15	5	1	23	3	0	0	0	0
Montevideo	29	8	0	0	0	1	0	0	0
Senftenberg	26	7	1	7	1	0	0	0	0
Typhimurium	8	8	2	1	0	0	2	0	0

### Calcofluor binding

**Figure 2:**
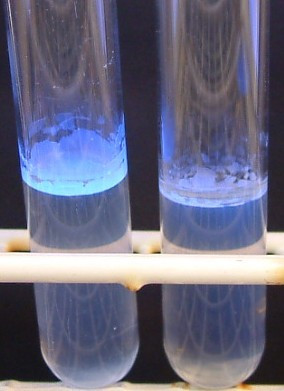
**Calcofluor binding in liquid-air interface (pellicle)**. Fluorescent pellicle under UV light indicates the presence of cellulose. Left: Calcofluor positive and Right: Calcofluor negative.

Screening of all 148 strains at least twice on both CF agar and on liquid revealed that 75% of the strains were consistent in Calcofluor binding, i.e. strains classified as rdar on CR agar always bound Calcofluor and strains classified as bdar and saw never did (Figure 2). *Salmonella *ser. Agona strains were found to be highly consistent with regards to Calcofluor binding or not, as 98% of all the strains were consistent. Inconsistency in Calcofluor binding was more widespread among the other serovars tested, with 13% inconsistent *Salmonella *ser. Montevideo strains, 17% *Salmonella *ser. Senftenberg strains and as many as 43% *Salmonella *ser. Typhimurium strains. This inconsistency was not dependent on systematic differences between the experiments, e.g. type of surface. Among strains classified as bdar on CR agar, no *Salmonella *ser. Agona bound Calcofluor, whereas over 60% of the other serovars were inconsistent, i.e. sometimes binding Calcofluor and sometimes not. When testing ten of the bdar strains (six *Salmonella *ser. Agona, two *Salmonella *ser. Senftenberg and two *Salmonella *ser. Typhimurium) on glass, steel and polystyrene, the same was observed, i.e. none of the *Salmonella *ser. Agona strains bound Calcofluor whereas the strains of the other serovars sometimes did.

As the *Salmonella *ser. Agona strains were consistent in Calcofluor binding, 13 strains classified as rdar and 13 strains classified as bdar of this serovar were tested in the crystal violet binding assay. Mean OD_595 _was 1.1 (SE = 0.1) in the bdar strains and 1.0 (SE = 0.1) in the rdar strains. No statistically significant difference between the two groups was observed (t test, p > 0.05).

### Polysaccharide analysis

**Figure 3:**
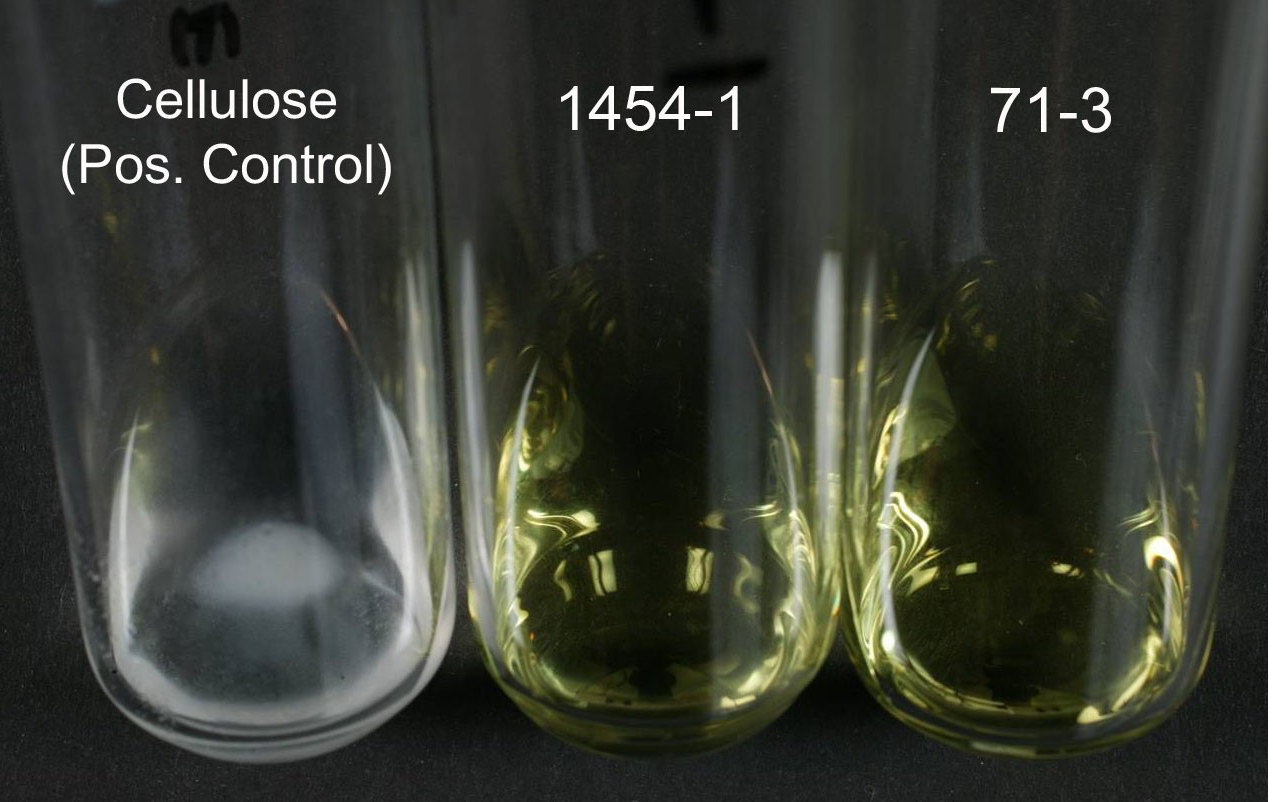
**Cellulose precipitation after isolation and treatment with acetic and nitric acid solution**. Left: positive control for cellulose production, Middle: *Salmonella *ser. Agona1454-1 (classified as bdar) and Right: *Salmonella *ser. Agona 71-3 (classified as rdar).

No visually detectable amount of cellulose was isolated from any pellicles or colonies derived from any of the *Salmonella *strains (71-3, 1454-1, ATCC 14028 and LT2) tested, and consequently no aqueous insoluble pellet was recoverable following treatment with the acetic acid-nitric acid mixture. No significant difference was found in the amount of glucose detected in the extracellular polysaccharides isolated from the biofilms of strain 71-3 (classified as rdar) and strain 1454-1 (classified as bdar) grown on agar whether the extracellular polysaccharides were treated with 12 M H_2_SO_4_, a pre-requisite for subsequent cellulose hydrolysis, or not (two-way ANOVA, p > 0.05, for all variables including interaction parameter). Cellulose was isolated from a positive control, i.e. the pellicle of *A. xylinum *confirming that the lack of detection of cellulose from *Salmonella *was not due to experimental error (Figure 3). Its identity was confirmed via its aqueous insolubility and resistance to hydrolysis in the acetic acid-nitric acid mixture. It comprised virtually pure glucose (> 95% w/w). Analysis of pure cellulose isolated as a positive control gave the same result.

Analysis of the biofilm polysaccharide isolated from colonies of the wild type *Salmonella *ser. Agona strains rdar 71-3 and bdar 1454-1 revealed the presence of rhamnose, mannose, galactose and glucose in a molar ratio of ca. 2:1:1:2 together with another unidentified monosaccharide. The polysaccharide material identified comprises about 25% w/w of the original dry biofilm. Analysis of the pellicle isolated from the two rdar strains 71-3 and ATCC 14028 revealed the presence of the same sugars in a molar ratio of ca. 1:1:1:2 respectively together with a smaller amount of arabinose. The polysaccharide material identified comprised about 15% w/w of the original dry pellicle.

#### The impact of rdar and bdar morphology on survival in biofilm

**Figure 4:**
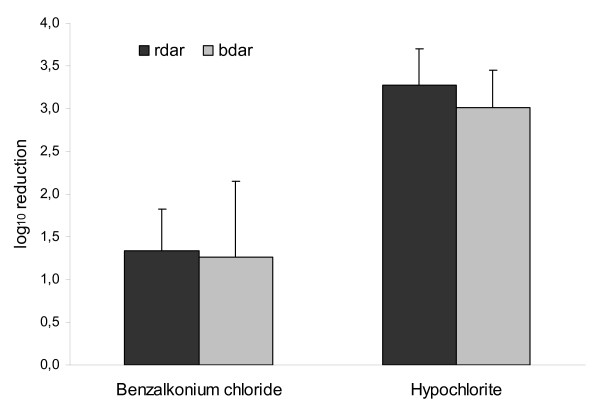
**Survival of *Salmonella *Agona strains with rdar and bdar morphotypes in biofilm after exposure to disinfectants**. Mean log_10 _reduction in total number of cfu in the biofilm after exposure to 0.05% hypochlorite or 0.02% benzalkonium chloride. Mean of three strains of each morphotype with standard error of the mean. Differences between the rdar and bdar strains are not statistically significant.

A statistically significant bactericidal effect of hypochlorite and benzalkonium chloride was found for both the rdar and bdar strains in biofilm (paired t test, p ≤ 0.05). Exposure to hypochlorite resulted in a 3.3 log_10 _mean reduction for the rdar strains and a 3.0 log_10 _mean reduction for the bdar strains. Exposure to benzalkonium chloride resulted in a 1.3 log_10 _mean reduction for both the rdar and the bdar strains (Figure 4). No statistically significant difference was observed between the rdar and the bdar strains when exposed to either hypochlorite or benzalkonium chloride (t test, p p > 0.05).

**Figure 5:**
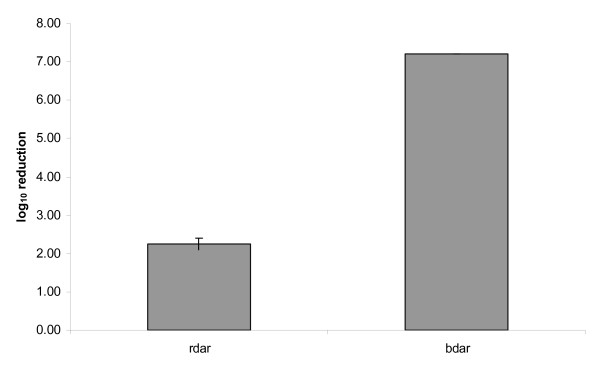
**Long-term survival of *Salmonella *Agona rdar (71-3) and bdar (1454-1) morphotypes in biofilm**. Mean log_10_reduction in total number of cfu in the biofilm after approximately four months of storage. Mean of two experiments with standard error of the mean (SE for bdar = 0.00).

The long-term survival potential of *Salmonella *rdar and bdar strains in biofilm was determined after approximately four months of storage at 20°C in a dry environment. This showed that the total number of cfu in the biofilm of strain 71-3, classified as rdar, was reduced by 2.3 log_10 _from an initial average log_10 _cfu of 10.6. The number of cfu in the biofilm of strain 1454-1, classified as bdar, was reduced by 7.2 log_10 _from an initial average cfu level of 9.3 log_10 _(Figure 5). The reduction was statistically significantly lower for the rdar strain than the bdar strain (paired t test, p < 0.05).

## Discussion

Little focus has earlier been given the bdar morphotype, probably because this morphotype has been believed to be relatively rare in nature. However, results from the present study show that as many as 55% of *Salmonella *ser. Agona strains displayed this morphology on CR agar. This percentage of bdar strains is high compared to other serovars, both in the present study and in other studies [11,12,18]. In a study by Solano *et al*., all bdar strains were of environmental origin, whereas Solomon *et al*. found approximately the same percentage of bdar among clinical strains and strains from produce (24 and 27%, respectively) [11,12]. In our study 61% of the *Salmonella *ser. Agona strains of feed factory origin and 38% of those of human clinical cases displayed bdar morphology, indicating that this is a general characteristic of this serovar independent of source of isolation.

Introducing Calcofluor to the LB broth allowed us to observe indication of cellulose expression on a variety of surfaces. Interestingly, the *Salmonella *ser. Agona strains were highly consistent in Calcofluor binding in our test systems. The strains classified as bdar on CR agar never bound Calcofluor on any of the surfaces tested, i.e. agar, liquid, glass, steel and polystyrene. In contrast, a high degree of inconsistency was observed within the other serovars. This may be due to a phenomenon earlier reported in some strains called high frequency switch (hfs) and low frequency switch (lfs), i.e. that cells from the same strain display different morphotypes [18].

Cellulose has earlier been reported to protect *Salmonella *against environmental stress [13]. It is therefore surprising that as many as 61% of the *Salmonella *ser. Agona strains of feed factory origin displayed the bdar morphotype on CR agar and did not bind Calcofluor, indicating no cellulose expression. *Salmonella *ser. Agona is a problem in the feed industry all over the world [29], and in some Norwegian feed factories, a number of clones have been shown to persist for several years [3,4]. Several of these clones display the bdar morphotype, indicating that strains with this morphotype are highly competent in surviving in this environment. Also in laboratory studies like the crystal violet binding assay, persistent strains of both bdar and rdar morphology were good biofilm producers and no statistical differences were found between the two morphotypes.

In the present study, we were therefore interested in potential differences between rdar and bdar morphotypes of *Salmonella *ser. Agona with regards to factors contributing to survival in the environment. It has been suggested that cellulose protects the bacterial cells against the bactericidal effects of disinfectants [11,13]. This is challenged by our results. We observed no significant differences in the susceptibility to hypochlorite or benzalkonium chloride when testing feed factory strains of rdar and bdar morphotypes. We have also observed similar effects in an earlier study using an alcohol based disinfectant and hypochlorite [30]. The discrepancies between our results and others' may be due to the fact that one of the other studies was performed on biofilms that had been kept several months in a very dry environment before testing, whereas the other study compared rdar cells in biofilm with bdar cells in suspension.

However, our results did show that the rdar strain tested was more tolerant to long-term desiccation and nutrient depletion than the bdar strain tested. Although it is difficult to know whether this result reflects a difference due to morphotype or a difference between single strains, the result is in accordance with those from a study performed by White *et al*. using *Salmonella *ser. Typhimurium ATCC 14028 and knockout mutants of this strain in order to determine the role of cellulose in long- term survival [13]. Both our laboratory experiment and the one reported by White *et al*. were performed under extremely dry conditions without any access to nutrients, whereas the feed factory environment is probably not constantly as dry, and the nutrients are more accessible. This may explain why bdar strains are still able to survive and persist in the factory environment despite our results showing that the bdar strain was less tolerant to long- term desiccation and nutrient depletion in biofilm than the rdar strain.

In our chemical analyses we could not identify cellulose in the matrix of any of the *Salmonella *strains tested, even though cellulose expression was indicated in the rdar strains by Calcofluor binding. However, Calcofluor is an extremely sensitive, down to the picogram range, and specific probe towards β-(1 → 4) linked D-glucopyranosyl residues which in the case of the extracellular matrix of bacteria is in form of cellulose [31]. Cellulose may therefore have been present in amounts below the detection level of the method used in our study. Other studies have also failed in detecting any substantial amounts of cellulose in the matrix of *Salmonella *rdar strains. Ariany *et al*. only found cellulose in the order of 100 - 150 ng in 200 mg of lyophilized cell mass of two wild type rdar strains (*Salmonella *ser. Typhimurium LT2 and DT104) [24]. Zogaj *et al*. showed cellulose in a *Salmonella *ser. Typhimurium 14028 pdar mutant by a methylation analysis of glucose biopolymers resulting in the exclusive identification of 1 → 4-linked glucose, but the amount of cellulose isolated per dry weight cell mass was not reported [32]. No cellulose could be directly visualized by atomic force microscopy in the matrix of ATCC strain 14028, but extracellular matrix was observed in the cellulose deficient mutant to almost the same extent as in the wild type [33]. Also the wild type rdar and bdar strains used in our survival experiments produced approximately the same total biofilm mass, as measured in the crystal binding assay, and approximately the same number of cfu; indicating that they had approximately the same amount of matrix. Based on all this information, we conclude that cellulose is not a major extracellular polysaccharide component of the *Salmonella *strains investigated under the culturing conditions used. Interestingly, despite the obvious low levels, cellulose still seems to play an important role in the structure of the biofilm. It has previously been shown that the rdar morphology is due to the expression of both fimbriae and cellulose contributing to a highly organised structure, and this organised structure is disrupted at the loss of one of these components [11,19,20]. Our hypothesis is that this structure might be of importance for the long term survival of *Salmonella*, whereas other bioflm components protect the bacteria against disinfectants like hypochlorite and benzalkonium chloride.

Curli fimbriae, the cell surface protein BapA (biofilm-associated protein), as well as an O-antigen capsule are other components known to contribute to the matrix [34,35]. In addition, an unknown matrix component has been reported in *Salmonella *rdar strains and their cellulose negative mutants. In bdar morphotype mutants of the *Salmonella *ser. Typhimurium 14028 strain, abundant extracellular matrix was observed by electron microscopy [32]. Transmission electron microscopy studies on a *Salmonella *ser. Enteritidis revealed an abundant, ruthenium red-positive matrix between bacteria in all samples, indicating the presence of negatively charged polysaccharides [11,13]. Analyses by White *et al*. (2003) indicated the presence of an anionic, extracellular polysaccharide distinct from colanic acid [20]. The monosaccharide composition of the major extracellular polysaccharide(s) we isolated in our strains seems in-line with other published works for *Salmonella *including *Salmonella *ser. Agona [36,37]. However, it is not possible to say whether this carbohydrate material is extracellular in the form of slime, as a capsule more tightly associated with the outside of the cell, or lipopolysaccharide which forms the basis of the outer leaflet of Gram negative outer membrane. Consequently, there does not appear to be major differences in polysaccharide content between the bdar and the rdar strains. Thus the wild type bdar strain does not have a major polysaccharide that is lacking in the rdar strains.

## Conclusion

In conclusion, the present study shows that *Salmonella *ser. Agona strains classified as bdar morphotype are commonly isolated. The *Salmonella *ser. Agona bdar strains are highly consistent in not binding Calcofluor, indicating no cellulose expression, in contrast to bdar strains of the other serovars studied. The *Salmonella *ser. Agona bdar morphotype was in this study found to be equally tolerant to disinfectant treatment as the rdar morphotype, whereas a *Salmonella *ser. Agona strain classified as rdar morphotype was found to be more tolerant to long-term desiccation and nutrient depletion in biofilm than a *Salmonella *ser. Agona strain classified as bdar. There does not appear to be major differences in polysaccharide content between the bdar and the rdar strains. Our results indicate that cellulose is not a major component of the biofilm matrix of *Salmonella *ser. Agona and *Salmonella *ser. Typhimurium, but the presence of even small amounts of cellulose contributes to a highly organised structure of the matrix.

## Authors' contributions

LKV was responsible for the study design, performing all screening experiments, experiments testing long term survival and effect of disinfectants on rdar and bdar strains, analysis of data from these experiments and preparation of the manuscript. LLN participated in all these parts. TM, SB and SL were responsible for establishing, performing and interpreting data from the polysaccharide analysis of matrix components. All authors contributed to the study design and revision of the draft manuscript. All authors have read, edited and approved the final manuscript.
